# MOH Drug SCM Strategy Development: a means to identify human resource training needs in Indonesia

**DOI:** 10.1186/2052-3211-7-S1-O4

**Published:** 2014-12-17

**Authors:** Engko Sosialina, Prihatiwi Setiati, Setiawan Soeparan, Russ Vogel

**Affiliations:** 1 Directorate of Public Medicines, Pharmacy Support DirJen, MOH, Sinapore; 2 Directorate of Public Medicines, Pharmacy Support DirJen, Ministry of Health, Jakarta, Indonesia; 3 USAID|DELIVER PROJECT, Ministry of Health, Jakarta, Indonesia; 4 USAID|DELIVER PROJECT, Jakarta, Indonesia

## Method

To develop the National Drug Supply Chain Management Strategy (SCM) Strategy, BinFar formed a strategy team made up of key SCM partners and staff. A drug SCM situation analysis was undertaken in 14 units within the MOH, exploring SCM issues and concerns, including staff development. Based on the results of the situation analysis, a stakeholders meeting was held to determine the core substance of the strategy and build a consensus on the key issues, including human resource development.

## Results

A national drug SCM strategy was developed to ensure drug availability, drug quality, and drug affordability for the public. The strategy framework included challenges and opportunities, a Vision, Mission and Goal statements, and developed strategy components based on the traditional SCM cycle. Based on the situation analysis results and in depth discussion with stakeholders, the strategy reviews each SCM cycle component regarding challenges and current practices and experiences. Based on these factors, a specific component strategy was developed with several strategic measures. It was concluded that human resource development was essential to support the entire drug management system.

## Discussion

To support the national drug SCM strategy, the following is needed:

1. Development of pharmacists competent as drug managers at all levels, including at health service facilities in hospitals and health centres.

2. Trained SCM personnel to ensure the smooth running of the drug management information systems.

The following strategies have been suggested to move this agenda forward:

1. Complete task analysis for every SCM competency required and develop pre-service and in-service training for pharmacists.

2. Stipulate a personnel standard for service locations that include pharmacists trained in SCM.

## Lessons learned

A national drug SCM strategy is a good opportunity to identify human resource SCM needs.

Obtaining consensus across programs and departments for a national drug SCM strategy requires strong baseline data and considerable dialogue.

Human resource issues and solutions are the backbone of a national drug SCM strategy.

